# Co-designing a general practice-led intervention and implementation strategy to increase bowel cancer screening through general practice: a qualitative study

**DOI:** 10.1186/s12875-025-03016-4

**Published:** 2025-10-31

**Authors:** Stephanie Walker, Kelera Levu, Melissa Tran, Corey Henshaw, Kate Broun, Glenn Austin, Lyndal Trevena, Natalie Taylor, Eleonora Feletto

**Affiliations:** 1 The Daffodil Centre, The University of Sydney, and Cancer Council NSW, Sydney, Australia; 2 School of Medicine, The University of Sydney, Sydney, Australia; 3 Cancer Council Victoria, Melbourne, Australia; 4 Queensland Health, Brisbane, Australia; 5 School of Public Health, The University of Sydney, Sydney, Australia; 6 School of Population Health, University of New South Wales, Sydney, Australia

**Keywords:** general practice, colorectal cancer screening, co-design, intervention

## Abstract

**Objective:**

Regular screening can reduce the burden of colorectal cancer (CRC) and general practice involvement has been demonstrated to improve screening uptake and CRC outcomes. Our study explores Australian general practice stakeholders’ current involvement in supporting organised CRC screening through the National Bowel Cancer Screening Program (NBCSP) and their views of the feasibility and applicability of evidence-based intervention components.

**Methods:**

A qualitative study was undertaken using focus groups (*n* = 12) with 45 participants that included General Practitioners (GPs), practice nurses, practice staff and program support staff from across Australia. The Consolidated Framework for Implementation Research (CFIR) was used as a guiding framework for deductive thematic content analysis.

**Results:**

The findings indicated that proactive GP involvement in the NBCSP is challenged by clinical information system limitations, time constraints, and competing priorities. Participants expressed a willingness to support CRC screening activities, understood the effectiveness of screening, but placed little focus on using the NBCSP as the primary method of CRC screening. An intervention to increase NBCSP participation involving a risk assessment tool, electronic reminder prompt, clinical information system and workflow enhancement, and education was viewed as feasible and applicable to practice if identified implementation challenges in the inner setting and individuals CFIR domains were addressed.

**Conclusions:**

General practice stakeholders acknowledge their potential for a more enhanced role in supporting the NBCSP and contributing to reducing the impact of CRC. A multi-component general practice-led intervention to promote an enhanced role was perceived as acceptable. To improve feasibility and applicability, the intervention needs to be integrated, straightforward, time-efficient, and supported by incentives and a whole-of-practice approach.

**Supplementary Information:**

The online version contains supplementary material available at 10.1186/s12875-025-03016-4.

## Introduction

Screening for colorectal cancer (CRC) is crucial to reducing disease burden and in improving population health outcomes [1]. Free immunochemical faecal occult blood test (iFOBT) screening has been provided to all Australians aged 50–74 years via the National Bowel Cancer Screening Program (NBCSP) and was phased in from 2006. However, NBCSP participation is currently only 40% [2] and it is estimated that reaching and sustaining 60% participation would save 84,000 lives between 2015 and 40 [3]. Recently, clinical guidelines were updated to include people from age 45 to 49 who will be able to opt into the NBCSP from 1 July 2024 [4]. These participation rates and guideline changes have spurred exploration into mechanisms that can improve CRC population screening participation internationally [5–17].

Successful evidence-based interventions include those targeting individuals, e.g. patient reminders [10], and the population, e.g. media campaigns [18]. Another avenue for intervention is through General Practitioners (GPs), which has been shown to improve CRC outcomes [5, 10, 19, 20] and to be acceptable to patients [21–23]. In Australia, GPs currently have limited direct NBCSP involvement as iFOBTs are mailed to the eligible individual. However, GPs are uniquely placed with the necessary clinical expertise to determine and endorse guideline-appropriate risk-based CRC screening, and their involvement can improve uptake [10, 24, 25].

GPs and their practice staff are willing to play a more active role in increasing CRC screening [6, 19, 26, 27] but do not always have the means. The evidence for effective general practice-led interventions to improve involvement in the screening pathway can be grouped into five broad categories of intervention components: risk assessment and decision support tools [28, 29], reminder prompts [30–32], clinical information system and workflow enhancement [33–38], general practice-focused education [19, 20, 34] and communication skills training [39]. Despite this evidence, many demands are placed on general practice as the health care front-line and the implementation of new interventions requires additional commitment, system-level changes, buy-in and behaviour change [27, 40–43].

Changing behaviour is challenging [41, 42], particularly in the high-workload general practice setting. The introduction of interventions beyond usual care must be feasible (realistically able to be integrated in the practice) and acceptable (have buy-in from the key stakeholders that will be involved in its delivery and use) to be successfully implemented. Relevant implementation factors may include contextual barriers and facilitators (e.g. competing practice priorities), stakeholder roles (e.g. varying levels of expertise and motivation) and intervention characteristics (e.g. compatibility with existing systems) [44]. Behaviour change techniques and implementation strategies tailored to general practice can address these factors and support implementation [14, 41, 44–47].

There have been some recent strategies launched to support general practice involvement in CRC screening through government health agencies and other bodies. A national digital platform was launched in 2020 which included the Health Care Provider Portal (part of the National Cancer Screening Register: NCSR Portal) which gives GPs direct access to their patients’ NBCSP screening history [48]. Local primary care organisations have supported implementation and continued NCSR Portal use through practice training visits and distribution of training resources and its impact on NBCSP participation is still being assessed [48]. Evidence-based interventions need to be brought together with current practice and support activities to determine what suits the needs of local general practice stakeholders. To do this, our study explores general practice stakeholders’ current involvement in supporting organised CRC screening through the NBCSP, their views of evidence-based intervention components, and their feasibility and applicability in the Australian context.

## Methods

A qualitative design was chosen to identify current barriers to GP involvement in the NBCSP (Additional File 1: Supplementary Table 1). Convenience and snowballing sampling techniques were used to recruit a target of 50 participants at which point data saturation was expected based on previous studies [49]. Advertisements were distributed through general practice networks, stakeholder newsletters and social media. Participants were eligible if they were a GP, practice nurse or staff member working within an Australian accredited general practice. Participants were also eligible if they were an employee of a health organisation supporting general practice to improve NBCSP participation (referred to as program support staff). The study excluded practices using paper-based patient records and solo-practicing GPs due to the need to explore the functionality of an intervention within software systems and reliance on a team-based approach [48, 50]. A gift voucher of AUD$200 was provided on completion to cover time costs of participation for GPs, practice nurses and staff [51]. The study was approved by the University of Sydney Human Research Ethics Committee (Approval: 2022/755). All participants completed an informed consent form in line with the ethics approval before taking part.

### Focus group setting

A series of 12 virtual focus groups (duration ranging from 60 to 90 min) were conducted between December 2022 and April 2023 by three independent investigators with one acting as facilitator and two observers providing technical support with prior knowledge of bowel screening. The first 10 focus groups were completed with GPs, practice nurses and staff in the evenings to accommodate work schedules and support national representation. A subsequent two focus groups were completed with program support staff during work hours. Focus groups were selected over individual interviews to facilitate dynamic interaction as well as the sharing of knowledge and information on this topic. Focus groups also allowed a larger group of participants to take part in the study. All were conducted online using MS Teams and recorded for transcription purposes.

Semi-structured and open-ended questions were used to guide all the focus group (see Additional File 2: Supplementary Material 1 & 2). Participants were asked about: (a) their current involvement in supporting the NBCSP, (b) challenges faced supporting the NBCSP within general practice, (c) preferences on categories of evidence-based intervention components (outlined in Table 1) based on a previously conducted scoping review (unpublished) to improve NBCSP participation, and (d) their views on the barriers and enablers to implementing the components in practice.

**Table 1:** Intervention components assessed in the focus groups

Intervention component category	Intervention Component examples
Risk assessment and decision support tools (28,29)	CRC risk assessment or decision support tool for GPs
	CRC risk assessment or decision support tool for patients
Reminder prompts (30–32)	Electronic point-of-care prompt
	Patient prompting GP with screening history
	Electronic waiting room prompt for practice staff
	Electronic reminder reports for GPs of patients due for screening
System and workflow enhancement (33–38)	Use of National Cancer Screening Register (NCSR) Portal and integration of NCSR Portal in practice clinical information system (CIS)
	Clinical auditing
Education (19,20,34)	Online learning modules
	Structured group workshops with bowel cancer screening educator
	Individual online or face to face training with bowel cancer screening educator
	Informational resources e.g. quick reference sheets, guides and brochures
Communication skills training (39)	Conversation guides/scripts and checklists
	In person or online communication skills training

Participants were presented with each intervention component and asked to discuss their use in practice. After the discussion, participants were asked to undertake a component ranking exercise to indicate their preferred component through an interactive online platform. An option of ‘other’ was included as a substitute for none of the above or to raise additional components. During the focus group, an investigator collated the online responses in real-time and presented the top-rated components for a final discussion of their feasibility and applicability. Program support staff were not asked to rank components but asked about their perceptions of the components’ feasibility and applicability based on the overall rankings from GPs, practice nurses and staff.

### Data analysis

Deidentified quantitative rating data were compiled and used to support the analysis. All focus groups were transcribed and deidentified by an independent investigator prior to analysis. Two investigators undertook a preliminary inductive thematic analysis after completion of all focus groups which identified current NBCSP involvement as well as barriers and enablers to the intervention components. The Consolidated Framework for Implementation Research (CFIR) was then used as guiding framework for deductive thematic content analysis. The CFIR comprises 39 constructs, categorised into 5 domains associated with implementation of interventions [44]. The framework was chosen to enable structured identification and interpretation of the components and factors (barriers and facilitators) that may affect implementation. Group discussions were undertaken to review analysis per the CFIR construct and define overarching themes. Subsequently analyses were conducted independently by four investigators using NVIVO. Two of the four investigators were not involved in the focus groups.

## Results

A total of 23 GPs, 15 general practice staff members (practice nurses and administrative staff) and 7 program support staff participated (Table 2), with representation from six Australian jurisdictions mostly from metropolitan areas (76%) at which point saturation was reached. Each focus group included a mixture of participants with all roles in the general practice with the exception of two focus groups which included only program support staff.

**Table 2:** Focus group participant characteristics

Characteristics	Number (*N* = 45)
Gender	
Male	4
Female	41
Role	
GP	23 **(2 = male)**
Nurse	7
Practice staff (e.g. manager, receptionist)	8
Program support staff	7 **(2 = male)**
State	
NSW	10
QLD	7
WA	2
VIC	4
TAS	1
SA	19
NT	2
Location	
Metro	34
Inner Regional	5
Outer Regional	4
Rural	2

### Current NBCSP involvement

Participants reported varied approaches to supporting the NBCSP. Proactive involvement included clinical audits, established recall and reminder systems, extended consultation times for preventive health checks and NBCSP waiting room promotions. These were described as ‘whole of practice’ activities performed by all:

*“The GPs that I work with are very meticulous with their use of the reminder system…as well as nurses…we do … GP management plans and screening is built into our GP management plan.” (Practice Nurse)*.

In comparison, opportunistic approaches were also commonly discussed amongst participants:

*“Our clinic does not do regular recall for patients to have bowel screening and we only do it when people come to us for other health issues” (GP)*.

Often GPs provide non-NBCSP iFOBTs (through private pathology). GPs commented on the ease, autonomy, and immediacy of action in issuing a referral:

*“I’ll just do private [non-NBCSP iFOBT] …Usually with the pathology form for their other routine bloods. And I find that’s a little more likely than me saying do you have the kit at home.” (GP)*.

Challenges reported by the participants included observed patient hesitance in discussing screening, stigma surrounding the cancer and screening, lack of patient interest or awareness of screening and lack of time and financial incentive to discuss screening in a standard consultation.

### Evidence-based intervention components

Participants discussed intervention components informed by their past experiences or their understanding of the intervention. For each intervention category, GPs, and general practice staff participants (*n* = 38) identified a top ranked intervention component or identified an alternative, more suitable component (Table 3).

**Table 3:** Top ranked intervention components by general practice participants (data collected from Slido)

Intervention Category	Top rated Intervention Component	Participant Preference (*n* = 38)
Risk assessment and decision support tools	CRC risk assessment or decision support tool for GPs	17
Reminder prompts	Electronic point-of-care prompt	14
System and workflow enhancement	Use of NCSR Portal and integration of NCSR Portal in practice clinical information system (CIS)	25
Education	Online learning modules	11
Communication skills training	Other*	17

*Other = defined by participants as either nothing or a factsheet of key messages to promote the NBCSP

Communication was the only component in which GPs ranked ‘other’ highly which meant that either they did not consider it a beneficial intervention or they considered a factsheet of key messages would be useful. However, practice nurses identified communication guidance for use with patients resistant to screening as beneficial. For other general practice staff, it was largely considered beyond the scope of their role.

### Component feasibility and applicability to practice

Overall, participants supported the implementation of the top ranked components. However, four implementation factors were highlighted which would need to be addressed to support intervention component feasibility and applicability in practice: 1) competing priorities and time scarcity, 2) incentivisation, 3) integration of systems, and 4) whole-of-practice approach. These focused on the inner setting domains of the CFIR [44], highlighting structural characteristics of general practice as well as relative priority of a new innovation and incentive systems to support implementation. The individual domain also influenced feasibility and applicability in terms of leadership and supporting for a new activity.

### Competing priorities and time scarcity

Participants noted that competing priorities and time scarcity during consultations impacted their ability to discuss the NBCSP and would influence the feasibility and applicability of any intervention:

*“Most people go to the doctor*,* and…they’re not looking for something else to talk about… they’ve already got a million things.” (GP)*.

Participants considered intervention components that were *“short and succinct and…simple to understand” (GP)* and digitally accessible to be feasible within the standard patient consultation time. For example, GP participants were willing to use a risk assessment tool if concise and accessible in the practice Clinical Information System (CIS):

*“If [a patient] asked me what [their] risk is*,* I would have to try and find a guideline…a template built into Best Practice [CIS] or a link to a web-based one… would be really useful.” (GP)*.

Participants expressed limitations in the process of ordering an NBCSP kit through the NCSR Portal (clinical system and workflow enhancement category). The time lag between ordering a NBCSP kit and its arrival at the home was an additional concern. Participants wanted to be able to provide an NBCSP kit at the point-of-care:

*“. it would be a lot easier for us to have [NBCSP] kits ready … if we were getting the form and the testing kit together and then giving it to the patient*,* we just hand them the box.” (GP)*.

GPs and nurses identified electronic point-of-care prompts as efficient but could result in over-prompting and ‘prompt fatigue’ which could diminish utility over the longer term. To maintain perceived benefit and mitigate ‘prompt fatigue’, participants preferred targeted prompts to be activated for patients at risk. Several participants recalled previous success in a monthly practice-wide “*focus on one particular issue at a time”.* This was viewed as a facilitator and a means to promote the NBCSP to both practice providers and patients *“because otherwise…it all gets very lost”* (Practice Staff) among competing priorities.

Intervention components that required a time commitment outside of consultations, such as education and clinical audits, competed with workflow priorities. Participants acknowledged their utility but were not convinced by the necessity and efficacy in practice:

*“I’m just not going to do an audit. I just haven’t got time…if someone did that all for me…that’s excellent*,* but it’s not going to happen from me.” (GP)*.

### Incentivisation

Participants agreed that a focus on prevention is an important priority, but more feasible if it *“translates directly to the thought of the [general practice] business” (Program Support Staff)* and the lack of financial incentive for deprioritised CRC screening:

*“We don’t screen for bowel cancer very systematically…If there was an incentive*,* then my boss [would] definitely… go forward here.” (GP)*.

Some participants recounted that non-financial incentives could facilitate prioritisation of the NBCSP. For example, receiving NBCSP rate reports could incentivise GPs to improve:

*“If I knew that the bowel cancer screening rates were much lower than I thought they should be*,* then I would put some time and effort into it.” (GP)*.

GPs and practice nurses engage in education and clinical auditing across a range of health topics for mandatory professional development. If these were aligned with the NBCSP and incentives, such as Continuing Professional Development Points (CPD), it would increase their priority:

*“Because of the changes to CPD now*,* where we’re having to do these…measured outcomes every year*,* I’m so excited to do a bowel screening audit because…I have to do one anyway” (GP)*.

### Integration of systems

Centralising information required during consultations from various digital platforms to the CIS was emphasised as beneficial integration and critical to facilitating the NBCSP. Participants viewed the CIS as the primary source of information and main interface to record actions. Components that integrated with their CIS such as the NCSR Portal, were identified as more feasible and applicable:

*“Having [the NCSR Portal] embedded in your practice software [CIS] is helpful because it’s just right there. We don’t have to open up something else. Yeah*,* it just makes things flow a little bit easier.” (GP)*.

Participants highlighted that the feasibility of reminder prompts and clinical audits, was dependent on CIS data quality and could make it difficult to accurately report screening history:

*“If it’s not coded in a specific way the audit software can’t read*,* it doesn’t recognise that [patient NBCSP history] exists.” (Program Support Staff)*.

Data quality and system integration were discussed as facilitators and participants viewed the NCSR Portal as a suitable component to access up to date information. However, those familiar with the NCSR portal, raised its limitations and called for additional training and education to support its use in practice:

*“But what I want the NCSR [Portal] to do …I want a permanent record to say this is what the NCSR told us about the patient. And then it also should record what we actually did about it.” (GP)*.

### Whole-of-practice approach

Participants supported a whole-of-practice approach to improving CRC screening, as this enabled shared motivation, relevance, responsibility, and expertise. Practice leaders with an enthusiasm for preventive health and ability to motivate others were identified as key facilitators to promote the intervention, as ‘practice champions’:

*“So having mutual education [on intervention components] from… champions in the practice…because within a practice*,* there are experts in various things.” (GP)*.

Participants suggested sharing the responsibility of implementing an intervention across staff to align with a whole-of-practice approach and leverage *“the capabilities of everyone inside the practice”* (Program Support Staff). The patient-facing role of GPs was viewed as essential to the feasibility of components reliant on clinical judgement, and that practice staff could support GPs by leading the non-patient-facing component of clinical auditing:

*“Our [practice] manager will capture…data and say this is how many patients we have that should be screened… and gives that sort of statistics of what we should be working towards” (GP)*.

Whereas minimal applicability was identified for practice staff involvement with components requiring clinical expertise such as the risk assessment tool, reminders and NCSR Portal:

*“So*,* I don’t really want to be engaging with patients as to [ask] have they had a bowel cancer screening… also just from a patient confidentiality perspective.” (Practice Manager)*.

Practice staff and nurses were identified as effective ‘practice champions’ and implementation leaders that can both educate and remind others to prioritise NBCSP practice initiatives. Participants also reinforced engagement of GPs as a crucial determinant of practice-wide success:

*“We sort of try to work with practices and create a proactive approach so that it’s a whole practice who’s working on it …if the GPs are not entirely involved*,* then there’s a bit of lag.” (Program Support Staff)*.

## Discussion

Study participants responded favourably to evidence-based intervention components to improve CRC screening. Overall, they supported CRC screening but did not always prioritise the NBCSP as the mechanism to provide CRC screening in their daily practice. The components assessed were familiar to participants but were not always viewed as feasible and applicable. Participants were receptive to a multi-component intervention that includes a CRC risk assessment tool, electronic point-of-care prompt, NCSR Portal integration with the practice CIS, and online learning modules (including fact sheets). These components were considered feasible and applicable if implementation considerations were addressed: competing priorities and time scarcity, incentivisation, integration of systems and a whole-of-practice approach.

The themes are consistent with previous research that has explored factors impacting general practice involvement in supporting the NBCSP [27, 43, 49]. Time pressure and suboptimal data integration across systems were previously identified barriers consist with our findings [27, 43, 49]. Previous studies have recognised that involvement of general practice in NBCSP initiatives should consider incentives, shared responsibilities across practice roles, and access to patient data within the CIS [27, 43, 49]. In our study, intervention components that were digitally accessible, simple to use and required minimal labour were preferable by practice staff. Additionally, implementation success could be improved by utilising collective skills and capacity of the practice workforce.

Views on the individual components highlighted important implementation considerations. For CRC risk assessment tools, there is evidence to support their use [28, 29], However, participants noted the lack of concise CIS-integrated tools available which limit their feasibility. Electronic point-of-care prompts are often used in practice and evidence shows that, for CRC screening, they provide a discussion prompt and can improve screening uptake [27, 30]. Participants supported the use of electronic prompts but flagged the possibility of ‘prompt fatigue’, suggesting targeting prompts to focus on key patient groups.

Evidence for clinical information system and workflow enhancement has demonstrated success in practice however is reliant on high quality and complete patient data [20, 33–35, 37]. Participants perceived NBCSP data quality and the time involved in clinical audits as a significant barrier but acknowledged the benefits of improved and integrated systems. Currently, the NCSR Portal provides a potential approach to address previously noted barriers [48]. Participants largely viewed this as a favourable component and were willing to learn more, especially relating to the utility of the NBCSP module of the NCSR Portal. The participants with experience using the NCSR portal identified a key barrier as the manual entry of data from the NCSR Portal into the practice CIS which could be overcome with automated data transfer now available in some practice CIS. Additionally, a recent report highlighted the inability to download practice-specific patient lists due or overdue for screening as a barrier [36].

GPs view their current involvement in the NBCSP as limited [27] which was reiterated by participants of our study. Australian evidence has identified the provision of NBCSP kits by GPs as a mechanism for their direct involvement [27]. Our study participants also suggested that NBCSP kit provision in-consultation as a positive way through which they could support the NBCSP. Consistent with findings of a recent evaluation report [34], some participants expressed concern over the current process to order NBCSP test kits compared to the familiarity and ease of non-NBCSP referrals. As of late 2023, the NBCSP has expanded the pathway by which participants can receive a kit, to allow GPs to provide a test kit directly to eligible patients, using the NCSR portal – the Alternative Access to kits Model (AAM) [7, 52]. The adoption of AAM as part of the NBCSP was informed by a pilot conducted in Indigenous communities and showed the AAM NBCSP participation was 39.8% in the study cohort [35].

Education is commonly used to support interventions in general practice [19, 20, 53]. Demanding workloads and requirements of GPs to participate in education for a range of health topics [54, 55], were common challenges [37, 56, 57]. These challenges were echoed by our participants to feasibility of education. Participants favoured self-directed online learning and concise modules aligned with incentives. A 2021 report also identified succinct, online education sessions as preferable [34]. Based on our findings, if NBCSP education is provided, flexible and short sessions with some incentive would be considered favourably by GPs.

Evidence has shown communication skills training for GPs can increase CRC screening [39]. However, communication was the only category not prioritised by our participants and opinions of the feasibility and applicability varied based on their role. GPs perceived communication skills as innate, not requiring additional training, whereas practice staff expressed that NBCSP-related communication was beyond the scope of their role. In comparison, practice nurses recognised the benefit in developing skills for communicating with patients adverse to screening. This is consistent with findings of a recent evaluation report in which GPs rated higher confidence than practice nurses for explaining CRC screening to their patients [20]. Our findings suggest that tailored communication support, for example factsheets, are considered feasible by practice staff.

The focus group methodology used allowed us to explore various perspectives on a single issue and enabled participants to build upon each other’s views [58, 59]. Our participants came from a range of stakeholder groups which gave us an understanding across roles within general practice and personnel requirements for each component. The open communication used in focus groups allowed participants to proactively engage with each other and individual experiences relating to the components were discussed enthusiastically. This resulted in peer-to-peer learning, an unintended benefit of our study, which is also a successful technique in supporting behaviour change and intervention implementation in Australian general practices [53]. Our study demonstrates that utilising the collective knowledge of all stakeholders offers a valuable approach to supporting interventions within the practice environment.

The study is not without limitations, which include the lack of representation across all general practice stakeholders and geographic locations. Deliberate efforts were made to recruit a diverse pool of participants from across Australia. While most jurisdictions were represented, the recruitment method employed may have resulted in selection bias favouring participants in active metropolitan regions, female participants and those with a greater interest in preventive care. This impacts the generalisability of the findings which may not fully reflect the experience in a wider Australian context by all general practices. While almost three-quarters of Australian’s health workforce is female, there are more male general practitioners than females [60]. This study had more female participants which may limit the generalisability of the findings. Though a quarter of the study participants were from outside metropolitan areas, no differences in components or implementation considerations were highlighted by the participants based on remoteness. It was not possible to conduct further analysis by subgroup to determine intervention variations for specific settings. As general practices become more involved in the NBCSP, intervention variations may become more suitable and future work could review the changing needs of general practice. The multi-component general practice-led intervention developed through this work has been trialled in Australia (ACTRN12623000355673 registered on: 5th April 2023). However, the focus of the study was on the applicability and feasibility of general practice-led interventions to daily practice from the general practice perspective rather than that of the patient. Future studies could explore patient experience, views and acceptability of these NBCSP interventions.

## Conclusion

General practice stakeholders acknowledge their current opportunistic role in promoting CRC screening and the potential for a more enhanced role in supporting the NBCSP. A multi-component general practice-led intervention to promote an enhanced role was acceptable. The intervention would include a risk assessment tool, electronic reminder prompt, system and workflow enhancement, and online learning modules and fact sheets. The feasibility and applicability of intervention components should be supported by implementation strategies and consider factors including integration within existing systems, time available in a consultation or the practice and support through incentives and a whole-of-practice approach.

## Supplementary Information

Supplementary Material 1

Supplementary Material 2.

## Data Availability

The data that support the findings of this study are held by the Research Team. Restrictions apply to the availability of these data, which were used under HREC requirements for this study. Data are available from the authors and subject to requesting permission from the University of Sydney Human Research Ethics Committee.
