# Supplementary material for: Co-designing a general practice-led intervention and implementation strategy to increase bowel cancer screening through general practice: a qualitative study
Source: BMC Prim Care. 2025 Oct 31;26:331. doi: 10.1186/s12875-025-03016-4 (PMC12577302; doi:10.1186/s12875-025-03016-4)
Supplement: Supplementary file 1 — Supplementary Material 1 [file 12875_2025_3016_MOESM1_ESM.docx]

**Additional File 2: Focus Group Guides**

**Supplementary Material 1 Focus Group Guide: *General Practitioners, Practice Nurses and Practice Staff)***

**Script:**

*Introduce topic*:

Welcome and thank you for joining this focus group. My name is XX and I am XX

*Welcome Slide:*

I’ll begin with the Acknowledgement of Country

We acknowledge and pay our respect to the traditional custodians of the lands and waters on which we work, and all Aboriginal Elders, past, present and emerging and their continuing cultural, spiritual customs and practices.

We work within The Daffodil Centre which is a joint venture of Cancer Council NSW and The University of Sydney. It is a leading research centre on cancer control and policy. This focus group will run for 90 minutes. We will be recording the discussion because we don’t want to miss any of your comments. A reminder that participation is entirely voluntary, and you have the right to withdraw at any point during the focus group without providing a reason. Whilst we are on a first name basis tonight, all responses are confidential, and no names will be used in any reports from focus group findings. You can share anything you want to - there are no wrong answers, please feel free to share your point of view even if it differs from what others have said.

This focus group is broken into three sections based on the project objectives.

During this session we ask that you use either the Microsoft Teams hand function if you would like to speak or ask a question. Alternatively, you can leave comments in the chat. We will also be using an interactive platform called slido. At times you need access to slido there will be a QR code on the powerpoint slide that you can scan using your phone.

*Project Overview Slide:*

Our project is about mobilising the National Bowel Cancer Screening Program by evaluating optimal interventions. Our focus today is Aim 3 – to design a general practice led intervention to increase bowel cancer screening participation. We would like to explore ways in which participation in the National Bowel Cancer Screening Program can be supported through your involvement.

*CRC Guidelines Slide:* Clinical guidelines exist to outline screening recommendations for individuals with different risk of bowel cancer.

We know that some individuals require specialised screening regimes inclusive of colonoscopies. Our focus today is individuals at near average risk that should be screening within the national program according to the guidelines.

*NBCSP Slide:* The National Bowel Cancer Screening Program could save 84,000 lives by 2040 if screening participation of near average risk individuals increased to 60%. The participation rate is currently 43.8%. Having a national program also allows a way to centrally track and monitor patients. We know that 80% of patients who participate in the program will re-screen when they are next invited.

*NBCSP Process Slide:* It is known that primary care providers, including GPs have a critical role in supporting bowel cancer screening. Formally the existing role of the GP starts when a patient receives a positive result and required further examination. Evidence suggests that it is beneficial to move the role of the GP earlier.

*CRC Screening Groups Slide:* The colorectal cancer burden in Australia remains high and there are many people who either do not screen at all or screen using colonoscopies when they do not need to. Our project is interested in how we can align screening behaviours with the current guideline recommendations. General practice and GPs are critical in this step.

*Pilot Interventions Slide*

This focus group will contribute to the design of a general practice led intervention to increase program screening participation. The intervention is intended to be trialled in 2023 within 80 general practices. The results of the trial will contribute to a National Interventions Scale-Up Plan.

*Objectives Slide:*

We would like to get your perspective on what components may form the best composition of the intervention. We would like to understand the potential barriers and enablers which are likely to impact on the uptake of intervention components.

*Process of interview:* I’ll begin by asking how you view your current involvement in the National Bowel Cancer Screening Program. I will then ask your opinions about different example intervention components. We will discuss the value of the intervention components in practice and any additional comments you may have.

*Ways to improve screening slide:*

When considering the questions – an intervention component is something that is done within general practice to improve screening.

An implementation strategy is how it is done and how it is supported in being done.

The intervention components and strategies we discuss today have been identified in an evidence review as being effective at increasing bowel cancer screening participation. The final intervention will be developed from both the evidence review and the co-design with general practice. This co-design will help understand what the future role of General practice in supporting the national program could look like.

If it is alright with you, I will turn the recorder on now… (TURN ON RECORDER)

| SECTION | QUESTION | PROBES |
| --- | --- | --- |
| 5 MINS | | |
| GP and Organization Context | Firstly, can I begin by asking each of you: your role, how long you have been working at your practice for and what is the size of the practice you work within? | GP, GP trainee, Practice nurse, practice manager.  Experience and years intended to work.  Relationship and degree of commitment to their organization/work. |
| 30 MINS | | |
| Current involvement in supporting NBCSP participation.  This section will be used to explore how General Practice:   1. Currently discuss CRC screening recommendations with their patients. 2. Perspectives toward existing initiative that have facilitate their involvement in the NBCSP | What is your practice currently doing to identify patients who eligible for bowel cancer screening and the National Bowel Cancer Screening Program? | **Follow up considerations:**  Which person in the practice is responsible for this?  How does your practice collect data about patient bowel cancer screening?  What tools/practices are used? Data extraction tools and clinical audits– PENCAT, POLAR  Do you share the clinical data with your PHN through a data extraction agreement?  How do you use the data? E.g. quality improvement audit, for recall and reminder systems, monitoring progress, practice progress reports. |
|  | **Today we will be using an interactive platform called Slido. I will indicate when to use Slido.**  **Please open slido either by going to slido.com and typing in the above code OR by scanning the QR code on screen.**  **Any answers you provide will be anonymous to others in the group.** | |
|  | **Please type any initial thoughts that pop into your head on Slido, you can type multiple answers:**  What are some challenges to discussing bowel cancer screening with your patients? | Competing patient health priorities.  Time, resources, practice priorities.  Knowledge, communication, education.  Are any of these challenges more difficult to manage?  **Follow up considerations:**  How have you managed aspects that are harder?  What resources or supports have you used that have made it easier? |
|  | **Can you please use the raise hand function on Microsoft Teams to indicate whether you have heard of the NCSR Health Care Provider Portal?**  How have you interacted with the National Cancer Screening Register’s Health Care Provider Portal in practice? | Patient screening status, ordering iFOBT kit.  Clinical software system integration  **Follow up considerations:**  What are functions of the portal that have been useful?  What functions of the portal have been difficult? |
| 50 MINS | | |
| Optimal design of intervention components and implementation strategies to support GPs in endorsing the NCBSP  This section will be used to explore GPs perspectives on:   1. What components will form the best composition of an intervention? 2. What factors may impact the effectiveness of the intervention? 3. What strategies can support the implementation of components? 4. What different methods of collecting data are used at a GP level? | Our team has conducted an evidence review to identify components of interventions that have been successful in increasing bowel cancer screening participation within general practice.  I’d like to share with you some examples of intervention components and discuss your opinions of these components and how they could form the composition of general practice led intervention. From the evidence, we have categorised the following 4 components to explore with you.  The components are:   - Reminder prompts - System and workflow enhancement - High-intensity education - Communication skills training   Please consider that these components may be used alone or combination with each other. Your perspectives will help us determine this. | |
|  | The first intervention component we will discuss is reminder prompts for the GP or practice staff.  This may take the form of:  Electronic prompts in clinical software flagging when a patient is due or overdue for screening.  Electronic reports distributed to the responsible GPs patients due or overdue for screening.  Electronic waiting room prompts in software for practice staff of eligible patients.  Patient completing a screening survey in the waiting room and presenting their GP with results during the consultation. | |
|  | **Question** | **Probing Questions (only if needed)** |
|  | **Please use the Microsoft Teams raise hand function to indicate if any of you already do the described component in your practice.**  If already using this component in practice – what works and what are the barriers?  In your role, how would you feel about using this intervention component? (If not part of current practice)  **Please use Slido to now rank your preference of reminder prompts within general practice based on how well they would meet your needs – consider if the component is appropriate in your practice and will it be well received.**  Based on Slido ranking: Why do you think this component is more important than another in the intervention design? Please use features of the component to explain. | How different is it from what you’re already doing now? E.g. time, scope, intricacy, number of steps, alignment with/departure from practice/processes  What aspects of your work would need to change to incorporate the component into your practice? |
|  | The next intervention component we will discuss is general practice system and workflow enhancement.  This may take the form of:  Utilizing cancer screening toolkits to perform data cleansing, clinical audits, and establishing uniformed coding within clinical software.  Integrating the National Cancer Screening Register within practice software and using the Health Care Provider Portal during patient consultations to order an FOBT via the portal. | |
|  | **Question** | **Probing** |
|  | **Please use the Microsoft Teams raise hand function to indicate if any of you already do the described component in your practice.**  If already using this component in practice – what works and what are the barriers?  In your role, how would you feel about using this intervention component? (If not part of current practice)  **Please use Slido to now rank your preference of system enhancements within general practice based on how well they would meet your needs – consider if the component is appropriate in your practice and will it be well received.**  Based on Slido ranking: Why do you think this component is more important than another in the intervention design? Please use features of the component to explain. | How different is it from what you’re already doing now? E.g. time, scope, intricacy, number of steps, alignment with/departure from practice/processes  What aspects of your work would need to change to incorporate the component into your practice? |
|  | The next intervention component we will discuss is education for the GP or practice staff.  This may take the form of:   - Individual online or face to face training tailored for GPs or practice staff - Online learning modules - Structured group workshops for the general practice | |
|  | **Question** | **Probing** |
|  | **Please use the Microsoft Teams raise hand function to indicate if any of you already do the described component in your practice.**  If already using this component in practice – what works and what are the barriers?  In your role, how would you feel about using this intervention component? (If not part of current practice)  **Please use Slido to now rank your preference of types of education within general practice based on how well they would meet your needs – consider if the component is appropriate in your practice and will it be well received.**  Based on Slido ranking: Why do you think this component is more important than another in the intervention design? Please use features of the component to explain. | How different is it from what you’re already doing now? E.g. time, scope, intricacy, number of steps, alignment with/departure from practice/processes  What aspects of your work would need to change to incorporate the component into your practice? |
|  | The final intervention component we will discuss is communication skills training for GPs or practice nurses. This may take the form of:   - The provision of conversation guides, scripts or checklists with strategies for raising a discussion about bowel cancer screening with non-compliant screening patients. - In person or online communication skills training with strategies to address barriers to screening and reasons for non-participation. | |
|  | **Question** | **Probing** |
|  | **Please use the Microsoft Teams raise hand function to indicate if any of you already do the described component in your practice.**  If already using this component in practice – what works and what are the barriers?  In your role, how would you feel about using this intervention component? (If not part of current practice)  **Please use Slido to now rank your preference of communication skills training within general practice based on how well they would meet your needs – consider if the component is appropriate in your practice and will it be well received.**  Based on Slido ranking: Why do you think this component is more important than another in the intervention design? Please use features of the component to explain. | How different is it from what you’re already doing now? E.g. time, scope, intricacy, number of steps, alignment with/departure from practice/processes  What aspects of your work would need to change to incorporate the component into your practice? |
|  | **We would now like for you to have the opportunity to provide any final comments on the top-rated intervention components.**  From the 4 intervention categories we have discussed today – these are the components you voted which you believe are your preference to include in a multi-component intervention.Please provide any considerations on the suitability of these components within practice. | |
|  | Any further considerations and close focus group. | |

**Supplementary Material 2: *Focus Groups Guide (Program Support Staff)***

**Introduction and informed consent:**

**Introduce topic:**

We would like to explore with representatives from local health organisations ways in which participation in the National Bowel Cancer Screening Program (NBCSP) can be supported through the increased involvement of general practice. This focus group will help us understand your current perspective on general practice involvement in promoting NBCSP participation and its flexibility to provide new services to increase participation.

**Define problem:**

The colorectal cancer (CRC) burden in Australia remains high and there are many people who either do not screen at all (under screened) or screen using colonoscopies when they do not need to (inappropriately screened). Under screened individuals are at higher risk of developing advanced stage bowel cancer, whereas inappropriately screened individuals can place unnecessary strain on the health care system, overcrowding colonoscopy waitlists.

**State purpose of focus group:**

This focus group will contribute to the design of a general practice led intervention to increase NBCSP participation. It forms part of a series of focus groups that are being held with GPs and general practice staff to understand their perspective on what components may form the best composition of the intervention and whether the components would be acceptable in practice. Importantly, we would like to know your views (from a health service perspective) on whether the co-designed intervention will be sustainable in the long term.

**Process of interview:** I’ll begin by asking what you view as current barriers to general practice involvement in the National Bowel Cancer Screening Program. I will then ask your opinions about different example intervention components. These components have been identified in an evidence review undertaken by our team as being effective in increasing CRC screening. We will discuss the value and implement-ability of the intervention components in practice, with a focus on how they might be sustained into the future, and any additional comments you may have.

Just a reminder that this discussion will be recorded. Participation is entirely voluntary, and you have the right to withdraw at any point during the focus group/interview without providing a reason.

If it is alright with you, I will turn the recorder on now… (TURN ON RECORDER)

| SECTION | QUESTION | PROBES | |
| --- | --- | --- | --- |
| 5 MINS | | | |
| Introduction | Welcome  Purpose of focus group  Focus group details: 60-90 mins, reimbursement, confidentiality  Consent | N/A | |
| 5 MINS | | | |
| Health organisation representative and Organization Context | Firstly, can I begin by asking each of you: your role, how long you have been working at your health organisation for and what is the size of the organisation you work within? | Representative role & position  Experience and years intended to work.  Relationship and degree of commitment to their organization/work.  Metropolitan/regional/rural location  Funding – ie. PHN (federally funded), department of health (state funded) or local government organisation (eg. ACCHO)  How many general practices does the organisation look after?  Does the organisation have an existing role in promoting CRC screening? | |
| 30 MINS | | | |
| Current perspective on general practice involvement in supporting NBCSP participation.  This section will be used to explore how representatives:   1. Currently view general practice involvement in the NBCSP 2. Consider how GPs can be better supports to endorse the NBCSP 3. What projects are ongoing to promote CRC screening activities in general practice? |  |  | |
|  | What are some challenges to general practice involvement in promoting CRC screening with their patients? | Competing patient health priorities.  Time, resources, practice priorities.  Knowledge, communication, education.  **Follow up considerations:**  How have you managed aspects that are harder?  What resources or supports have you used that have made it easier? | |
|  | What is your health department currently doing to encourage general practices to support patients with CRC screening? | Quality improvement initiatives (PIP QI)  Community education and CRC screening campaigns  Practice facilitation  Funding for recall and reminder systems (eg. GoShare)  Existing or planned initiatives?  **Follow up considerations:**  Will the pilot intervention impact on existing initiatives or vice versa?  How does your organisation collect data about patient CRC screening?  What tools/practices are used? Data extraction tools and clinical audits– PENCAT, POLAR/primary sense  Do you have access to general practice data through a data extraction agreement?  How do you use the data? E.g. quality improvement audit, for recall and reminder systems, monitoring progress, practice progress reports. | |
|  | How have you observed general practices interacting with the National Cancer Screening Register’s Health Care Provider Portal in practice? | Patient screening status, ordering iFOBT kit.  Clinical software system integration  **Follow up considerations:**  What are functions of the portal that have been useful?  What functions of the portal have been difficult? | |
| 10 MINS | | | |
| Optimal design of intervention components and implementation strategies to support GPs in endorsing the NCBSP  This section will be used to explore perspectives on:   1. What components will form the best composition of an intervention? 2. What factors may impact the effectiveness of the intervention? 3. What strategies can support the implementation of components? 4. What is the role of local health organisations in supporting roll-out of the intervention? 5. What is important to considering regarding how the intervention will be able to be sustained in the long-term, post-trial completion. | Our team has conducted an evidence review to identify components of interventions that have been successful in increasing CRC screening participation.  I’d like to share with you some examples of intervention components and discuss your opinions of these components and how they could form the composition of a general practice led intervention.  **** Present example/s****  (e.g. point of care prompts, GP reminders, practice staff training). | | |
|  | **40 MINS** | | |
|  | **Question** | **Probing Questions (only if needed)** |  |
|  | Do you think this component would work in general practice and what would be the barriers if not? | How different is it from what’s already being used now?  What does this intervention add that is new? doing now? E.g. time, scope, intricacy, number of steps, alignment with/departure from practice/processes | |
|  | How important do you think it is to include certain components in the intervention design in comparison to one another?  Please use features of the components to explain. **(Skip if information already discussed)** | How do you think these components compare to other alternatives that could be considered or that you know about? | |
|  |  |  | |
|  | What organizational goals/priorities at your workplace would these components align with?  **(Skip if information already discussed)** | What do you see as the role of jurisdictional health organisations in supporting implementation of the intervention?  Would implementing these components help achieve these priorities?  Would they conflict with any other priorities? | |
|  | What roles internally and externally would need to be involved to implement these components? | Do you think that local health organisations have capacity to support implementation of the intervention?  What would need to change so that local health organisations have capacity to support implementation of the intervention? Does this differ between different jurisdictions (e.g different states of Australia) and how might this impact on intervention delivery? | |
|  | What kind of training would you or others need to implement any of the components discussed? | Improving data entry and extraction  Clinical guideline updates | |
|  | How would the resources available in your organization impact your ability to implement these components?  **(Skip if information already discussed)** | Funding for cancer screening activities  Dedicated staff members | |
|  | Form a jurisdictional, health department perspective, how do you feel about the sustainability of this intervention component? | Do you think this intervention is likely to be adopted by health departments following completion of the pilot? Why/why not?  Do you think this intervention is likely to be funded by health departments following completion of the pilot? Why/why not? | |
|  | Any further considerations and interview close | | |
